# A framework for building comprehensive cancer center’s capacity for bidirectional engagement

**DOI:** 10.1007/s10552-023-01848-y

**Published:** 2024-02-25

**Authors:** Erika S. Trapl, Sarah Koopman Gonzalez, Kristina Austin

**Affiliations:** 1 Department of Population and Quantitative Health Sciences, Case Western Reserve University, Cleveland, OH USA; 2 Case Comprehensive Cancer Center, Case Western Reserve University, Cleveland, OH USA

**Keywords:** Community engagement, Cancer center, Mixed methods, Frameworks

## Abstract

**Purpose:**

Community engagement has benefits for cancer centers’ work and for its researchers. This study examined the experiences and perceptions of community engagement by members of the Case Comprehensive Cancer Center (Case CCC) to create and implement a framework to meet the needs of the entire cancer center.

**Methods:**

This study included three phases: 1) Semi-structured interviews with 12 researchers from a basic science program to identify needs and suggestions for the support of community engagement; 2) Preliminary interview results informed the development of a survey of 86 cancer center members’ about their awareness of and readiness to integrate community outreach and engagement into their research; and 3) The Case CCC Office of Community Outreach and Engagement reviewed the results from phases 1 and 2 to develop and then utilize a framework of engagement opportunities.

**Results:**

In the interviews and surveys, cancer center members recognized the importance of community engagement and expressed an interest in participating in COE-organized opportunities for bidirectional engagement. While participation barriers include communication issues, limited awareness of opportunities, and competing priorities, members were open to learning new skills, changing approaches, and utilizing services to facilitate engagement. The framework outlines engagement opportunities ranging from high touch, low reach to low touch, and high reach and was used to develop specific services.

**Conclusion:**

This study identified varying needs around community engagement using an approach aimed at understanding the perspectives of a community of scientists. Implementing the framework enables reaching scientists in different ways and facilitates scientists’ recognition of and engagement with opportunities.

**Supplementary Information:**

The online version contains supplementary material available at 10.1007/s10552-023-01848-y.

## Introduction

The National Cancer Institute has demonstrated its intention for community engagement to be incorporated into cancer centers since formalizing community outreach and engagement as a required component of cancer center support grants since 2017. This was further supported through its 2020 initiative for “NCI P30 Cancer Center Support Grants to support community outreach and engagement (COE) activities across the translational research continuum.” Bi-directional community engagement can increase the relationship between catchment areas and cancer centers [1] and ensure that the work of cancer centers addresses community priorities [2].

There are additional benefits for researchers engaging with the community, as identified in other research studies. This includes early researchers being trained in community engagement, increasing scientists’ ability to communicate with community members creating sustainable partnerships, and ensuring the equitable impact of their research [3–5]. Additionally, community engagement can provide research motivation, inform the full research process, increase the uptake of findings, support the development of dissemination plans for grants, and assist with securing additional funding [3, 5, 6]. Bi-directional community engagement is also benefiting for community members, including providing opportunities for informing science and its dissemination, reducing mistrust, and increasing capacity [3].

There is a need to develop strategies to assist scientists in cancer centers with community engagement as catchment area research is relevant for all fields of science and not only for population science researchers [7]. While research has shown that basic scientists engage with the community less than other researchers [1], there are also barriers that impact scientists at all levels of the cancer research continuum [8, 9]. These barriers include identifying partners [3], lack of trust [3], competing priorities and time needed for engagement [3, 8, 9], as well as institutional fiscal and administrative barriers [3, 8, 9].

While previous research has described some benefits and barriers to engagement, little work has been done demonstrating examples of how to address barriers, particularly with basic scientists. This study aimed to understand the experiences and perceptions of, as well as suggestions for community engagement by members of the Case Comprehensive Cancer Center (Case CCC), to develop community outreach and engagement activities that meet the needs and interests of scientists. Like the work by George et al. [3], this study identified opportunities for the Case CCC Office of COE based on interviews and surveys conducted with Case CCC members. As a result of the memberships’ responses, we created a framework and described specific examples of how this framework can be used to meet the identified needs and interests within the context of the Case CCC.

## Methods

Our study was implemented in three sequential phases, with each subsequent phase building upon the prior phase. In Phase 1, we conducted semi-structured interviews with members of a basic science research program. Based on the themes gleaned from these interviews, we created a survey that was implemented with all members of the Case CCC. Finally, in Phase 3, we reviewed all data from Phase 1 and Phase 2 to inform a framework to create a range of opportunities for understanding the needs of the Case CCC catchment and facilitation bidirectional engagement of Case CCC researchers and community members.

### Phase 1: semi-structured interviews data collection and analysis

Interviews were conducted with 12 researchers who are members of one of the basic science programs in the Case Comprehensive Cancer Center between January and April of 2021. These interviews aimed to identify themes around perceptions, motivations, barriers, and capacity-building needs to inform strategies to support engagement in COE. Twelve interviews were completed allowing for thematic saturation [10]. Participants were identified across all experience levels (e.g., Assistant Professor, Associate Professor, Professor) as diverse in academic rank and career level (early career, mid-career, and senior level) and gender among interviewees. Before the interview, participants were sent the informed consent form via email, and participants verbally consented before starting the interview. Two researchers conducted the interviews via phone or Zoom video conferencing (*Zoom Video Communications*), which lasted about 45 min. Participants were asked questions about their engagement with individuals who have experienced cancer and with community members, such as the Community Advisory Board (CAB) of the Case Comprehensive Cancer Center. Additionally, participants were asked about the impact of their research on the community or local catchment area of the Case Comprehensive Cancer Center and their knowledge of how engagement can impact the catchment area. Basic scientists were the focus of this data collection phase, as across the translation continuum, they are the least likely to engage community stakeholders in their research [5].

Interviews were transcribed verbatim using Zoom software (*Zoom Video Communications)* or by the researcher who conducted the interview for interviews conducted over the phone. All transcripts were verified for accuracy before analysis. Transcripts were explored to identify topics, including needs and suggestions that could inform strategies developed by the Case CCC Office of COE. As part of the inductive thematic analysis [11], author SKG coded the interviews to identify participant suggested needs, suggestions, and perceptions and determined when themes had reached saturation. All authors met throughout the analysis to discuss findings and potential engagement opportunities to meet needs. The Case Western Reserve University Institutional Review Board approved all interview procedures.

### Phase 2: readiness survey data collection and analysis

Preliminary themes identified from the Phase 1 interviews were used to inform the selection of measures for a survey of cancer center members’ awareness of and readiness to integrate community outreach and engagement into their research. For example, in the interviews, we learned that many interview participants did not know about the Case CCC Community Advisory Board. As a result of this finding, the item “I am aware of the Case CCC Community Advisory Board and its role in Community Outreach and Engagement” was added to the survey. Many of the survey items were drawn from a tool that was shared by another comprehensive cancer center. The survey was reviewed by the research team and shared with other collaborators in the cancer center before distribution to the Case CCC membership. Full and Associate cancer center members (i.e., independent faculty members engaged in cancer research) of all six research programs of the Case CCC were invited to participate in the survey. Programs include cancer genomics, cancer imaging, developmental therapeutics, immune-oncology, molecular oncology, and population and cancer prevention. The survey was open for three weeks in January 2022; participants provided consent electronically before completing the web-based survey. The Case Western Reserve University Institutional Review Board approved the protocol for the survey with Case CCC members. Survey measures included the following domains:

#### Community outreach and engagement and its role within the case CCC

Participants were asked to indicate their agreement on a four-point scale from Strongly Disagree to Strongly Agree or select “I don’t know” to several items assessing the participants’ understandings and perceptions of Community Outreach and Engagement and its role within the Case CCC. Items included “I understand what the Case CCC COE team does” and “I am aware of the Case CCC Community Advisory Board and its role in Community Outreach and Engagement.”

#### Community outreach and engagement and my research

Several items were included in the survey to assess participants’ understanding of COE and their research, including “My research can be enhanced by directly engaging with community stakeholders” and “I know how to reach out/where to go to start engagement with the community.” Participants indicated their agreement on a four-point scale from Strongly Disagree to Strongly Agree but were also provided a fifth answer option of “I don’t know.”

#### Readiness for community partnership

Items to measure participant readiness for community partnership included, “I am open to learning new skills and behaviors for community outreach and engagement” and “I am open to changing my research approach or plan if another approach better serves community partners.” Participants indicated their agreement on a four-point scale from Strongly Disagree to Strongly Agree or could select the item “I don’t know.”

#### Interest in services

Participants were asked to review and indicate the services they would consider utilizing by answering yes, no, or maybe to potential services in each of the following domains: community input services, data from the community, and data about the community. The list of services was developed based on looking at similar shared resources at other comprehensive cancer centers.

In addition to these domains, participants identified their research program membership and prior experience with community engagement (personal or professional).

Descriptive statistics of survey results for all participants were conducted to assess the percentage of respondents that either somewhat agreed or strongly agreed with the items assessing community outreach and engagement and its role with the Case CCC, community outreach and engagement and their research, and readiness for community partnership. The percentage of participants who answered “yes” to indicate the services they would consider utilizing was also examined. We also examined responses for our 5 programs which house basic scientists (cancer genomics, cancer imaging, developmental therapeutics, immune-oncology, molecular oncology) and compared these to our population and cancer prevention program which houses nearly all of our population scientists to assess where there were similarities and uniquenesses in perspective and needs.

### Phase 3: framework and service development

The results from both the Phase 1 qualitative and Phase 2 quantitative data were examined by the Office of COE separately and developed a list of primary recommendations. After synthesizing the six recommendations and reviewing the results from Phases 1 and 2, the Director of COE, supplement investigators, and the authorship team developed an engagement framework which a system of ideas to plan and achieve a goal. This framework was used to modify current engagement activities and develop specific services across all framework areas to address scientists’ needs, gaps, and interests to engage with the local catchment area. These activities were sorted and depicted across the two dimensions of touch and reach, with the ideal to maximize at least one dimension; while high reach and high touch would be the ideal, these approaches may be overly resource intensive and not feasible. For example, activities in the “high touch, low reach” category have more significant, intensive interaction, and engagement (high touch) between scientists and community partners. However, the activity is limited to a smaller number of scientists and community partners due to resource constraints (low reach). Activities in the “low touch/ high reach” category involve less time and level of engagement from the scientists but reach the broadest group of individuals in the Cancer Center.

## Results

### Phase 1: semi-structured interviews

Interview participants spanned academic rank and career levels, including one trainee, two in their early career, four in their mid-career, and five at a senior level. Table 1 provides a summary of the qualitative themes and corresponding exemplary quotes from the interviews.

**Table 1:** Qualitative Themes and Exemplary Quotes

Theme	Exemplary quote
Importance of engagement	“I think the community interaction brings a different, it’s what I said before, it’s a different perspective on what’s important, you know.”
Engagement types and needs	“I’m not sure how you really get engagement without actually giving people more exposure and a, and an ability to build relationships within the community. It just sort of, it happens, it’s over and it just moves on to something else, and then, that’s, that’s the end of it. And we go back to our laboratories and that’s how it happens.” “So, I guess like lectures are pretty impersonal so that, that’s…You really have to engage your whole audience. So, I think, you know, small group meetings between, you know, one scientist and three to four people, that’s probably the most effective. And so those opportunities are rare. I guess, you know, that the larger types of lectures are more common but they’re less engaging to the audience.”
Barriers to engagement: communication	“I’ve seen it first hand at some of these events, someone will walk in and just they will talk the same way they talk to a colleague in the exact sub-specialty as they would to a community member, and you can imagine the outcome there.” “I would say, it’s always intimidating to try and take what we do and try to, present it in a way that’s compelling for others to understand why it’s important. It’s really difficult.”
Barriers to engagement: competing priorities	“… the drive for what we do is all a lot of things right, and some of its progress, you know getting paper out some sort of getting a grant funded or all these things that are sort of related. Um, and I, you know, I think that’s maybe one of the challenges, too, is that in this profession, we start to you know, chase money and get papers published and kind of lose sight of the big picture.”
Barriers to engagement: Limited Awareness	“I just, I don’t know what opportunities exist other than sort of informal. And maybe that’s my own ignorance, you know, admittedly. I just don’t, I’m not familiar with those.” “I think it’s the same thing. You know we just can’t find each other. Nobody essentially, I don’t think people tell them, you know, oh come and talk to our scientists.”
Barriers to engagement: understanding integration	“I think that would be very valuable for a lot of basic scientists that are just really unaware of, you know, how patient data could integrate with their own research program.”
Solutions for barriers: communication	“I think that’s the most important thing in terms of outreach to the community, especially non-scientists in the community is making them see where they fit into what you’re talking about.” “If you want this communication to work then somebody has to teach us how to do it.”
Solutions for barriers: competing priorities	“We have the retreats and call it so…From my perspective, calling it a retreat or something like that sounds less official and therefore less important on a CV. So I think the more that we can kind of bolster the importance of this community outreach conference […] Me saying that I won an award at a local retreat holds a lot less water than saying I won an award at this National Academy of Physicians Conference, right? They’re both an award and a scientific conference, but one of them is deemed more important because of where it came from, and just kind of the nomenclature of it.”
Solutions for barriers: limited awareness and understanding integration	“But, but I haven’t found that direct connection myself as well, too, because for me I’ve just sort of been focused on [disease] because of the funding we have. If there were opportunities, I think, to serve the community or to identify things for particular cancers here in our community, that would be something that I would look into.”

#### Importance of engagement

Engaging with the community was considered helpful and important for basic scientists and community members. Participants noted that community engagement helps with finding a common language, providing feedback on research, giving researchers a perspective of a disease they may not have, and providing benefits, such as learning opportunities for the community. With this, communicating with community members was described as important.

#### Engagement types and needs

While most participants described formal presentations to the community when discussing engagement, other types of engagement were also mentioned, including written communication, lab tours, radio and social media communication, workshops, educational events for community members, and having a panel of patients to learn from. Besides formal communication and presentations, participants also discussed wanting to have informal discussions and meetings with smaller groups of community members. These informal opportunities were described as more accessible for conversation and communication. They were also described as leading to relationships being built. Relationship building and “engagement outside of science” were defined as things that help with building partnerships and science. A few interview participants noted that these informal opportunities are not common.

#### Barriers to engagement

Although community engagement was seen as important, interview participants discussed barriers to engagement, including communication barriers, competing priorities, lack of awareness or access to opportunities, and limited understanding of how to integrate community into their research. Participants discussed how they and others find it challenging to develop messages for and communicate science with community members. Another aspect of communication that participants mentioned as complex is conveying the importance of basic science research to community members. There is a perception that community members are interested in treatments and the translation of research. Since basic science is not at that stage, it takes time to communicate such findings. Another barrier mentioned by interview participants is the competing priorities for their time and effort due to the various priorities and requirements for scientists in their roles. Some described how, although they are interested in engaging with the community, their time is limited. Participants discussed this as a barrier to participation in engagement activities and a common issue for other scientists. Limited awareness of opportunities or access to engagement opportunities was also described as a barrier. Some participants also stated that they needed to be aware of opportunities if they existed. Access and knowledge of opportunities were also described as barriers for community members. Relatedly, participants discussed that some basic scientists, including themselves, need more understanding of integrating their research with the community or catchment area.

#### Solutions for barriers

While participants described communication barriers, some discussed how they learned strategies from mentors and through trial and error. Some participants suggested offering training to learn and practice communication skills, which they noted they needed or other scientists could use. Additional suggestions made by participants around communication included having personnel available for assistance with communicating with community members, a mechanism for feedback to the scientist after an event or presentation, and providing examples of successful message delivery and communication.

While several participants described the barrier of competing priorities, few discussed potential solutions. A few mentioned communicating the value of engagement, having leaders identify this as a priority, and changing the names of meetings. One participant suggested changing the name of meetings and retreats that include community engagement to “conferences.” This interview participant felt that using a different terminology would look better on a resume and lead to prioritized engagement.

Participants also discussed the need for assistance understanding how their work can be engaged and related to the catchment area. There is an intersection between barriers and needs mentioned by participants, such as understanding how research connects to the community, what opportunities exist, and funding opportunities to address competing priorities. Overall, participants expressed interest in community engagement opportunities, learning skills for community engagement, and modifying their work for community engagement and benefit.

### Phase 2: readiness surveys

86 of the 421 members (20.4%) of the Case CCC participated in the survey, with responses equally represented across research programs. Survey respondents were primarily in the later stages of their careers, with more responses from full professors (43.8%) and a smaller response from associate (27.5%) and assistant professors (20.0%). Only three survey respondents were pre-doctoral trainees. Responses were predominantly from members with a PhD (58.0%). Only 57.5% of respondents were civically engaged.

Assessment of responses by each scientific program enabled identifying where each program may have unique needs compared to the overall membership. Not surprisingly, our population science program showed the greatest awareness and capacity for community engagement. However, our basic science programs were enthusiastic about engaging and building their skills and understanding for future engagement opportunities.

Survey respondents indicated the value of engagement as 85.6% of respondents agreed that their research could be enhanced by directly engaging with community stakeholders (with population and cancer prevention research program respondents at 94.8% agreement and the range of responses from the other five basic and translational science programs at 75.0–84.6% agreement).

Only 38.6% of survey respondents agreed that they know how to reach out or where to go to start engagement with the community, indicating a lower capacity for COE. The responses by research programs ranged from 63.2% agreement from population and cancer prevention research program respondents and between 23.1 and 41.6% in the other five basic and translational science programs.

Participants responses to the survey also expressed their interest in community engagement as well as making changes to their current processes; 93.9% of survey participants agreed that they are open to learning new skills and behaviors for community outreach and engagement (range of 94.8% in population and cancer prevention research program and 76.9% to 100% in the other five basic and translational science programs) and 79.5% indicated that they are open to changing their research approach or plan if another approach better serves community partners (range of 100% in population and cancer prevention research program and 61.6% to 100.% in the other five basic and translational science programs).

Additionally, when asked in the survey about interest in varying services, between 37.0 and 63.4% of participants expressed interest in each service type (Table 2). Of note, while only 63.4% of participants indicated that they would consider utilizing services for catchment area data, 85.5% responded that they agree with the statement “I am interested in learning more about the catchment area of Case CCC” (range of 94.7% in population and cancer prevention research program and 61.6–100% in the other five basic and translational science programs).

**Table 2:** Interest in community outreach and engagement services

Services	Overall % Yes would consider utilizing (*n* = 86)	Population and cancer prevention research program % Yes would consider utilizing (*n* = 19)	Range in five other research programs % Yes would consider utilizing (*n* = 11–13)
Community input			
Community conversations: 1–2 h facilitated discussion with community groups	37.0	42.1	16.7–46.2
Community advisory board presentation	56.8	63.2	46.2–60.0
Community/patient engagement studios: evidence-based, facilitated 2-h interactive retreat with community partners	45.7	42.1	23.1–58.3
Community readiness assessment: evidence-based qualitative approach to assess community readiness	39.5	47.4	23.1–50.0
Case CCC scientific research champion: connection to a non-academic research champion	50.6	52.6	23.1–66.7
Data from the community			
Survey design consultation	39.0	47.4	23.1–58.3
Focus groups	45.1	47.4	23.1–58.3
Data collection consultation	49.4	63.2	23.1–66.7
Study recruitment	53.1	63.2	23.1–75.0
Data about a community			
Catchment area data	63.4	78.9	38.5–75.0
Connection to community partners	50.6	52.6	15.4–75.0
Connect with a community research liaison: faculty or staff member with community research expertise	56.3	57.9	15.4–83.3
Community advisory board presentation	63.0	63.2	53.8–75.0
Dissemination	55.6	52.6	46.2–66.7
Presentation development	54.3	52.6	46.2–58.3
Provide topic expertise	59.3	63.1	46.2–75.0

### Phase 3: framework for community outreach and engagement opportunities

**Fig. 1:**
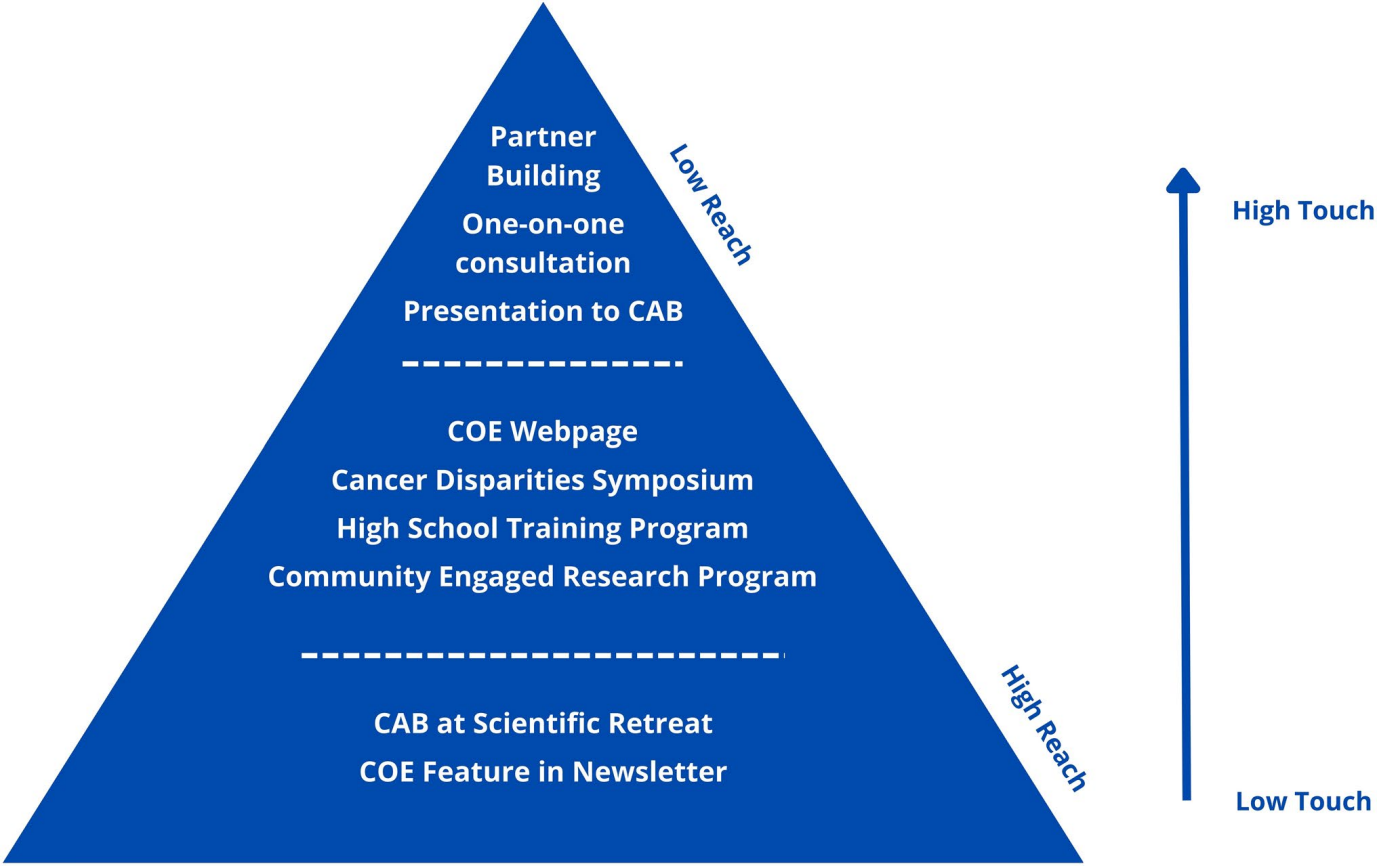
Framework of COE Opportunities

Based on the results identified in Phase 1 and Phase 2, the Case CCC Office of COE identified six primary recommendations: (1) Establish a COE Internal Advisory Board to assist in developing COE priorities to support CCC research programs; (2) Integrate CAB members into CCC activities; (3) Make catchment area data readily available to all CCC members via the COE website; (4) Expand opportunities for CCC members to present and engage with community partners; (5) Establish and promote COE consultations services for CCC members; and (6) Create and promote opportunities for non-transactional engagement. With these recommendations in mind, we developed a framework for community outreach and engagement opportunities from “high touch/low reach” to “low touch/high reach,” as depicted in Fig. 1.

One of the first initiatives implemented was establishing an Internal Advisory Board (IAB) for the Office of COE, consisting of one leader from each scientific research program. The IAB helps prioritize bidirectional initiatives to engage scientists and the community and serves as an additional liaison between cancer center members and the Office of COE. In collaboration with the IAB, we identified existing and new strategies based on the responses from the survey to facilitate scientist and community partner relationship building.

Existing COE strategies were modified to include our Community Advisory Board (CAB), Scientific Retreats, and the Cancer Disparities Symposium. The CAB was established in 2012 to connect Case CCC community partners with research program scientists to inform the community partners about current research and obtain feedback. The CAB’s current membership includes cancer survivors, individuals representing federally qualified health centers, the American Cancer Society, community cancer support centers providing free psychosocial support to individuals coping with cancer, and other community organizations interested in cancer and health disparities. The CAB meets six times per year. The COE team redefined engagement with the CAB by ensuring that each scientist presenting their work did so in a manner that was accessible to the lay members of the CAB and included a specific ask of the CAB to help scientists benefit from the CABs’ lived experiences and expertise regarding the respective communities they represent. The Office of COE, with guidance and input from the CAB, put together a procedure to guide the CCC member presentation to ensure that the presentation would facilitate bidirectional engagement. CCC members meet with the Office of COE before the presentation and answer the following questions to guide the development of their presentation: (1) Introduce yourself as a person; (2) What’s the ‘big problem’ your research is trying to solve? (3) What’s the specific question you are working on right now? (4) When you have the answer to this question, what happens next; how will it affect outcomes? (5) Beyond questions that the CAB will have, what questions do you have for the CAB as far as implications or dissemination of findings?

At the Case CCC Annual Scientific Retreat, each session is co-facilitated by a CCC scientist and a CAB member, and the CAB member poses questions from a community lens. The CAB’s role in the Scientific Retreat has broadened to include interacting with the members of each research program during a lunchtime breakout session to discuss the impact of community engagement and strategies to increase bidirectional engagement.

New activities by the Case CCC Office of COE include building individual partnerships with community members and organizations, one-on-one community partner consultations with scientists, and a COE spotlight in the CCC weekly newsletter.

The Case CCC Office of COE is exploring models to expand its cadre of community partners, including cancer survivors and caregivers, who are interested in being research partners. Case CCC COE intends to implement a Research Advocate program, where cancer survivors and caregivers are recruited and provided with Research 101 and Cancer 101 training. Once community partners have completed training, they can partner with a scientist and become a research team member. The community partner provides lived experience and perspective often absent from research projects. Implementing such a program at Case CCC will enhance our opportunity to support the growing capacity and interest of Case CCC scientists who want to integrate COE principles into their work and benefit from bidirectional engagement.

## Discussion

This study demonstrates the importance of understanding what scientists want to know and what they need assistance with, as community engagement has varying needs, capacities, and interests. To create opportunities that facilitate community engagement, the approach undertaken in this study was to understand the perspectives of a community of scientists in a similar way as we would approach understanding communities outside of academia. While we started with interviews with basic scientists, as research has shown that they engage with the community less than other scientists [1], we also surveyed the full cancer center membership to understand their interests and needs as there are barriers to community engagement faced by all scientists [8, 9]. It is crucial to examine what scientists want to know about community engagement and the catchment area and understand what they need assistance with to develop effective strategies for community engagement. In this study, similar themes were found in the surveys with all members as from the interviews with basic scientists, particularly around the importance of community engagement and barriers to engagement, such as lack of knowledge of opportunities.

Similar to other studies [3, 8, 9], this study found that time and competing priorities are barriers to community engagement. This study also showed how engagement and its importance can support other scientists’ priorities and meet their goals and interests. In response to understanding the specific needs and interests of the Case CCC, this study aimed to develop solutions that can address these needs and meet the local catchment area context. To do this, we created a framework for community engagement opportunities that would meet the different needs of scientists and incorporate community engagement into the fabric of the cancer center. This framework allows for reaching scientists at various time points and in different ways, from high-touch, low-reach opportunities that may assist with relationship building to low-touch, high-reach opportunities that provide a broader understanding and recognition of opportunities.

While we had a strong engagement in our Phase 1 interviews, participation in our survey was limited, with a response rate of just over 20%, despite our best efforts to distribute the survey through the CCC newsletter and emails from Program Leaders. This low rate could bias our results if we were concerned about the overall generalizability to our cancer center. However, if we shift our perspective to interpret the interview and survey results through a slightly different lens, we could interpret these responses as being gleaned from individuals across all programs who do have an interest in COE. Those responses become highly useful in designing a COE program for members already primed to take advantage of such efforts. In other words, we captured responses from our most activated audience. Gains in bidirectional COE are most likely successful when the opportunities are most accessible for the most motivated individuals.

Solutions that may decrease or eliminate barriers to scientists engaging with the community have been identified in other research, such as training scientists [1, 3, 4], training community partners [3, 9], starting engagement early in the research process [3, 9], and creating forums for discussions between patients and scientists [3, 4]. We argue that this framework can assist with the development of multi-level solutions to barriers that meet the local needs and context within their catchment area.

Although this study presents findings and the early development of opportunities based on the proposed framework, the framework has been used at one cancer center. It has yet to examine changes in perceptions of, readiness for, and participation in community engagement opportunities. However, we present an approach and framework that other cancer centers can use to identify opportunities that will best meet the needs of their members. The following steps in this study are to develop metrics to measure community engagement within this framework that can be used across cancer centers.

### Supplementary Information

Below is the link to the electronic supplementary material.

Supplementary file1 (DOCX 15 KB)

Supplementary file2 (DOCX 22 KB)

## Data Availability

Deidentified datasets can be made available by request to the corresponding author.

## References

[1] Leader AE, Aplin AE (2021). From the community to the bench and back again: the value of patient and community engagement in cancer research. Cancer Discov.

[2] Vadaparampil ST, Tiro JA (2022). Catchment area: an opportunity for collective impact, strategic collaboration, and complementary focus. Cancer Epidemiol Biomarkers Prev.

[3] George S, Vassar SD, Norris K, Coleman B, Gonzalez C, Ishimori M, Morris DA, Mtume N, Shapiro MF, Lucas-Wright A, Brown AF (2019). Researcher perspectives on embedding community stakeholders in T1–T2 research: a potential new model for full-spectrum translational research. J Clinical Translational Science.

[4] Ketcher D, Bidelman A, Le LQ, Otto AK, Lester DK, Amtmann-Beuttner KK, Gonzalez BD, Wright KL, Vadaparampil ST, Reblin M, Lau EK (2021). Partnering patients, caregivers, and basic scientists: an engagement model that fosters patient- and family-centered research culture. Translational Res.

[5] Kost RG, Leinberger-Jabari A, Evering TH, Holt PR, Neville-Williams M, Vasquez KS, Coller BS, Tobin JN (2017). Helping basic scientists engage with community partners to enrich and accelerate translational research. Acad Med.

[6] Holzer JK, Ellis L, Merritt MW (2014). Why we need community engagement in medical research. J Investig Med.

[7] Paskett ED, Hiatt RA (2018). Catchment areas and community outreach and engagement: the new mandate for NCI-designated cancer centers. Cancer Epidemiol Biomarkers Prev.

[8] Ahmed SM, Neu Young S, DeFino MC, Kerschner JE (2019). Measuring institutional community engagement: adding value to academic health systems. J Clin Transl Sci.

[9] Carter-Edwards L, Grewe ME, Fair AM, Jenkins C, Ray NJ, Bilheimer A, Dave G, Nunez-Smith M, Richmond A, Wilkins CH (2021). Recognizing cross-institutional fiscal and administrative barriers and facilitators to conducting community-engaged clinical and translational research. Acad Med.

[10] Guest G, Bunce A, Johnson L (2006). How many interviews are enough? An experiment with data saturation and variability. Field Methods.

[11] Braun V, Clarke V (2006). Using thematic analysis in psychology. Qual Res Psychol.

