# Supplementary material for: A framework for building comprehensive cancer center’s capacity for bidirectional engagement
Source: Cancer Causes Control. 2024 Feb 25;35(6):963–71. doi: 10.1007/s10552-023-01848-y (PMC11130016; doi:10.1007/s10552-023-01848-y)
Supplement: Supplementary file 1 — Supplementary file1 (DOCX 15 KB) [file 10552_2023_1848_MOESM1_ESM.docx]

Semi-Structured interview guide

Background:

Could you tell me a little bit about your training and professional background?

How long have you been doing research?

(if applicable) How long have you been a faculty member?

What is your career-stage?

What is your discipline?

What is the disease focus of your work?

[Probe for details about how long has been involved in cancer-related research, type of research done previously (if applicable), current role in the MO program]

[ other potential probes - How did you become interested in cancer research, how did you become involved in the Cancer Center]

Learning from the Experience of Others

What direct experience have you had with patients or individuals living with cancer?

Have you had any experiences where you got to learn from cancer patients about what their experiences have been with having cancer and pursuing treatment?

[Probe for discussions with patients, patient advocates, and interactions with community members]

If yes: Were any of those experiences from individuals living in the local area?

What did you think about those experiences?

How have those experiences influenced the work you do?

If no experiences: What experiences do you think would be valuable for you?

Have any of the experiences you’ve been describing been around the type of cancer you research?

What would you like to know about living with cancer that might help enhance your research?

How do you think your research impacts the life of a person with cancer?

Catchment Area:

As you probably know, NCI-designated Cancer Centers have a responsibility to conduct research relevant to and leveraging the features of their relevant catchment areas. What does that mean to you?

How would you describe the Catchment area of the Case Comprehensive Cancer Center?

[Probe for population/statistics and exposures related to cancer]

If you wanted to learn more about the catchment area, where would you go?

For each resource:

How did you learn about that resource?

Have you gone to that resource before?

If yes, what did you find?

What did you think about what you found?

How did you use what you found?

If no, in what circumstances would you go?

If says doesn’t know where to go: Under what circumstances would you start to look for more information about the catchment area?

How do you think you would use that information?

What about the catchment area would you want to know more about?

Could you tell me more about what makes you want to know more about those things?

How would you describe the relevance of your work for the catchment area?

Do you feel like our catchment area is particularly burdened by the type of cancer you research? (maybe something about cancer-related disparities as well somewhere?)

What would you want to know in order to increase the relevance?

When do you or would you need to describe the relevance of your work for the catchment area?

[Probe for grant applications, presentations, and discussions.]

Community Advisory Board

Have you ever met with the Case Comprehensive Cancer Center Community Advisory Board before?

If yes, could you tell me about that experience?

[Probe for specifics about where the interaction was, what was included in the interaction]

How did that experience impact you?

How did that experience influence your work?

If no – what about Advisory Boards from other institutions, or from other research interests?

If has an experience with other community advisory boards: Could you tell me about that experience?

[Probe for specifics about where the interaction was, what was included in the interaction]

How did that experience impact you?

How did that experience influence your work?

Have you known others that have met with the Case Comprehensive Cancer Center Community Advisory Board before?

If yes, what did you hear about that experience?

How do you think meeting with the Community Advisory Board would influence your work?

What do you think they would be interested in knowing about your work?

Would you have any concerns about the questions they might ask or feedback they might provide?

How often do you think basic scientists should meet with a community advisory board?

What do you think are some reasons why scientists might not meet with a community advisory board this often?

For reasons mentioned: What do you think could be done to help with that?

What do you think is the role of a CAB and a CAB member?

What responsibilities do they have to the community they represent as well as to the researchers?

What types of interactions with the community and community members do you want to have in relation to your work?

How do you think that type of experience would affect your work?

What do you think those experience would be like for community members?

How often do you think these experiences should happen?

Communication:

How would you describe your research to someone who is not in your field or not a scientist?

Have you ever described your research to someone who is not in your field or not a scientist before?

If yes, what was that like?

What do you think someone with XXX cancer (specific type to interviewee) would want to know about your research?

Barriers and Suggestions for interactions:

What do you think are some barriers to basic scientists interaction with the community?

For barriers mentioned: What do you think could be done to help with that?

What do you think would assist basic scientists in their interactions with the community?

What do you think are some barriers for community members meeting with basic scientists?

For barriers mentioned: What could be done about that?

What do you think would assist community members in their interactions with the community?
