# Supplementary material for: A framework for building comprehensive cancer center’s capacity for bidirectional engagement
Source: Cancer Causes Control. 2024 Feb 25;35(6):963–71. doi: 10.1007/s10552-023-01848-y (PMC11130016; doi:10.1007/s10552-023-01848-y)
Supplement: Supplementary file 2 — Supplementary file2 (DOCX 22 KB) [file 10552_2023_1848_MOESM2_ESM.docx]

**Community Outreach and Engagement and its role within the Case Comprehensive Cancer Center (CCC)**

|  | Strongly disagree | Somewhat disagree | Somewhat agree | Strongly agree | I don’t know |
| --- | --- | --- | --- | --- | --- |
| I understand what the Case CCC Community Outreach and Engagement team does. |  |  |  |  |  |
| I am confident I can describe the catchment area of the Case CCC. |  |  |  |  |  |
| I am interested in learning more about the catchment area of the Case CCC. |  |  |  |  |  |
| I am aware of the Case CCC Community Advisory Board and it's role in Community Outreach and Engagement. |  |  |  |  |  |
| Case CCC considers community outreach and engagement as integral to all cancer research. |  |  |  |  |  |

**Community Outreach and Engagement and My Research**

|  | Strongly disagree | Somewhat disagree | Somewhat agree | Strongly agree | I don’t know |
| --- | --- | --- | --- | --- | --- |
| I understand what community outreach and engagement means as it applies to my research. |  |  |  |  |  |
| My research can be enhanced by directly engaging with community stakeholders. |  |  |  |  |  |
| My funding opportunities can increase by engaging with community stakeholders. |  |  |  |  |  |
| I am apprehensive about working directly with the community. |  |  |  |  |  |
| Community outreach and engagement has no relevance to my research. |  |  |  |  |  |
| I understand what I can bring to a partnership with the community. |  |  |  |  |  |
| I understand what the community can bring to a partnership with scientists. |  |  |  |  |  |
| I know how to reach out/where to go to start engagement with the community. |  |  |  |  |  |

**Readiness for Community Partnership**

|  | Strongly disagree | Somewhat disagree | Somewhat agree | Strongly agree | I don’t know |
| --- | --- | --- | --- | --- | --- |
| I am apprehensive about incorporating community outreach and engagement into my research. |  |  |  |  |  |
| I am open to learning new skills and behaviors for community outreach and engagement. |  |  |  |  |  |
| I will listen to community stakeholders and others to enhance basic science data dissemination efforts into the community. |  |  |  |  |  |
| I am open to changing my research approach or plan if another approach better serves community partners. |  |  |  |  |  |
| I am ready to identify common goals and objectives with community partners. |  |  |  |  |  |

**Assessing Our Readiness to Act on Community Partnership**

Think about other members of the Case Comprehensive Cancer Center to answer the next set of items.

***Members of the Case Comprehensive Cancer Center:***

|  | Disagree | Somewhat disagree | Somewhat agree | Agree | I don't know |
| --- | --- | --- | --- | --- | --- |
| Are committed to implementing community outreach and engagement strategies in their research. |  |  |  |  |  |
| Are confident that the Case CCC will support investigators in adopting community outreach and engagement strategies in their research. |  |  |  |  |  |
| Are confident that they can handle the challenges that might arise from implementing community outreach and engagement in their research |  |  |  |  |  |
| Are motivated to implement community outreach and engagement strategies in their research. |  |  |  |  |  |

**Community Partners and Audiences**

How important are each of the following groups to your research?

|  | Not at all important | Somewhat important | Very important |
| --- | --- | --- | --- |
| Health care providers |  |  |  |
| Faith communities |  |  |  |
| Racial/ethnic minority communities |  |  |  |
| Sexual/gender minority communities |  |  |  |
| Workplaces or Employers |  |  |  |
| Patients |  |  |  |
| Family Members of Patients |  |  |  |
| Cancer Survivors |  |  |  |
| People with increased risk for cancer |  |  |  |
| Public officials and policy makers |  |  |  |
| Civic groups |  |  |  |
| Funders |  |  |  |
| Case CCC members in other programs |  |  |  |

There are many pathways to incorporate Community Outreach and Engagement (COE) to enhance research. Below is a list of COE Services that could potentially be offered to the Case CCC membership. Please indicate which services you would consider utilizing.

Community Input Services

|  | Yes | Maybe | No |
| --- | --- | --- | --- |
| Community Conversations: 1-2 hour facilitated discussion with community groups to gather information and/or explore topic prior to writing a grant |  |  |  |
| Community Advisory Board presentation: present research ideas or progress to receive feedback and questions from members who represent the catchment area |  |  |  |
| Community/Patient Engagement Studios: evidence-based, facilitated 2-hour interactive retreat with community partners to gather specific input on a funding proposal or active project |  |  |  |
| Community Readiness Assessment: evidence-based qualitative approach to assess community readiness to engage in an issue; usually includes 6-10 key informant interviews. |  |  |  |
| Case CCC Scientific Research Champion: connection to a non-academic research champion such as a community organization member, cancer survivor or caregiver who has experience with the population or disease. |  |  |  |

Data from the Community

|  | Yes | Maybe | No |
| --- | --- | --- | --- |
| Survey Design Consultation: survey design, measurement selection, data collection protocols and processes, recruitment methodology |  |  |  |
| Focus Groups: design, participant recruitment, and focus group facilitation |  |  |  |
| Data Collection Consultation: identify existing data sources and community partners, tailor tools for population, assist with implementation of data collection, access to community research liaison |  |  |  |
| Study Recruitment: consultation, connection to existing resources, share best practice and recruitment/retention strategies specific to population |  |  |  |

Data about a Community

|  | Yes | Maybe | No |
| --- | --- | --- | --- |
| Catchment Area Data: catchment area data, including disease incidence, mortality and late-stage diagnosis; risk behaviors that contribute to cancer risk; demographic characteristics and social determinants of health |  |  |  |
| Connection to Community Partners: help develop engagement plan; identify partners/organizations; provide introductions; assist with partnership agreements |  |  |  |
| Connect with a Community Research Liaison: Faculty or staff member with community research expertise and connection to a network of community partners |  |  |  |

Sharing Research with the Community

|  | Yes | Maybe | No |
| --- | --- | --- | --- |
| Community Advisory Board Presentation: present research and receive feedback and questions from members who represent the catchment area |  |  |  |
| Dissemination: Assistance with strategies; feedback on creating community-friendly materials; help identifying and connecting with appropriate community audience; training on communication techniques |  |  |  |
| Presentation Development: Training and resources to create and execute community presentations |  |  |  |
| Provide Topic Expertise: Be connected with community partners that might benefit from the expertise of the researcher; facilitate establishing partnership |  |  |  |

Now we’d like to know a little bit more about you so that we can tailor our COE programming efforts in the coming year.

What Research Program are you a member of?

- Cancer Genomics
- Cancer Imaging
- Developmental Therapeutics
- Immune Oncology
- Molecular Oncology
- Population and Cancer Prevention

How would you prefer to receive professional development about community outreach and engagement? Choose all that apply.

- Webinar
- One-on-one meetings/consultation
- Reading materials
- Newsletter
- Group Training

What is your terminal degree?

- MD
- MD/PhD
- PhD
- Other _____________________________

What is your current position/rank?

- Pre-doctoral Trainee
- Post-doctoral Trainee
- Assistant Professor (or equivalent)
- Associate Professor (or equivalent)
- Professor (or equivalent)
- Other _____________________________

Do you have experience working with the community?

- Yes, professionally and personally
- Yes, professionally only
- Yes, personally only
- No, I do not have experience with the community
